# Invasive Plants and Enemy Release: Evolution of Trait Means and Trait Correlations in *Ulex europaeus*

**DOI:** 10.1371/journal.pone.0026275

**Published:** 2011-10-14

**Authors:** Benjamin Hornoy, Michèle Tarayre, Maxime Hervé, Luc Gigord, Anne Atlan

**Affiliations:** 1 Ecobio, Centre National de la Recherche Scientifique, Université de Rennes 1, Rennes, France; 2 BIO3P, Institut National de la Recherche Agronomique - Agrocampus Ouest, Université de Rennes 1, Rennes, France; 3 Conservatoire Botanique National de Mascarin, Saint-Leu, La Réunion, France; University of Tartu, Estonia

## Abstract

Several hypotheses that attempt to explain invasive processes are based on the fact that plants have been introduced without their natural enemies. Among them, the EICA (Evolution of Increased Competitive Ability) hypothesis is the most influential. It states that, due to enemy release, exotic plants evolve a shift in resource allocation from defence to reproduction or growth. In the native range of the invasive species *Ulex europaeus*, traits involved in reproduction and growth have been shown to be highly variable and genetically correlated. Thus, in order to explore the joint evolution of life history traits and susceptibility to seed predation in this species, we investigated changes in both trait means and trait correlations. To do so, we compared plants from native and invaded regions grown in a common garden. According to the expectations of the EICA hypothesis, we observed an increase in seedling height. However, there was little change in other trait means. By contrast, correlations exhibited a clear pattern: the correlations between life history traits and infestation rate by seed predators were always weaker in the invaded range than in the native range. In *U. europaeus*, the role of enemy release in shaping life history traits thus appeared to imply trait correlations rather than trait means. In the invaded regions studied, the correlations involving infestation rates and key life history traits such as flowering phenology, growth and pod density were reduced, enabling more independent evolution of these key traits and potentially facilitating local adaptation to a wide range of environments. These results led us to hypothesise that a relaxation of genetic correlations may be implied in the expansion of invasive species.

## Introduction

Biological invasions are a major threat to global biodiversity, and thus many studies aimed at identifying species that are potential invaders and habitats that are prone to invasion. If some characteristics of invaded environments have been identified, such as a high level of disturbance or low species diversity [1], [2], no general biological properties of invasive species were identified [3], [4]. One explanation is that the spreading phase of an invasion process is habitat-specific so that invasive success is associated with different traits in different environments [1], [5]. Another explanation is that it is not the nature of life history traits but their capacity to adapt to a novel environment that is relevant. Indeed, the evolutionary potential of introduced populations is often considered a key factor in their invasiveness [6], [7]. This is supported by the observation of rapid evolutionary changes in many invasive species (e.g. in allelopathic chemicals, reproductive phenology or vegetative growth) in response to new biotic interactions or abiotic conditions [8], [9], [10].

Biotic interactions are always different in the native and introduced ranges, partly because exotic plants have usually been introduced without their pathogens, parasites or herbivores [2]. This release from natural enemies has been proposed as one of the most important ecological factors contributing to the invasiveness of numerous species [11], [12], [13]. Arguments on this Enemy Release Hypothesis (ERH) have been reviewed by Keane and Crawley [14] and Liu and Stiling [15]. Based on this release from natural enemies, an evolutionary mechanism was proposed by Blossey and Nötzold [16]: the Evolution of Increased Competitive Ability (EICA) hypothesis states that, because a plant has limited resources to invest in defence against enemies, growth and reproduction, an exotic species in an environment devoid of natural enemies will evolve to invest less in defence and more in other fitness components. The resulting increase in vegetative growth and/or reproductive effort would result in a better competitive ability of the species. This hypothesis makes clear and testable predictions: genotypes from the invaded regions should (i) be less well defended against natural enemies than genotypes from the native regions, and (ii) grow faster and/or produce more seeds.

The EICA hypothesis has been tested on many invasive plant species. It seems to play an important role in species such as *Silene latifolia* [11] and *Sapium sebiferum* [12], but in many others, evidence for increased susceptibility to natural enemies or increased growth and reproduction were not found [17], [18]. A shift in resources from defence to growth and/or reproduction is expected only if these defences are costly and directed against specialist enemies [5], [19], [20]. Moreover, the traits usually measured to test the EICA hypothesis (growth, reproduction, resistance to natural enemies) are often genetically correlated with one another or with other life history traits [21], [22]. This may prevent the evolution of traits predicted by the EICA hypothesis because the rate of trait evolution is affected by the respective intensity and direction of genetic correlations and selection [23]. More generally, genetic correlations can constrain the local adaptation of ecologically important traits such as vegetative growth and flowering phenology, and hence prevent further range expansion of introduced species [24]. In studies that aim to compare plants from native and invaded regions, it might thus be important to compare not only the trait means, but also the correlations among traits. Yet, due to the release from natural enemies in the introduced regions of the species, we might expect a relaxation of the strength of genetic correlations between defence against these enemies and life history traits, potentially leaving these life history traits more free to evolve during invasion.

The common gorse (*Ulex europaeus*, Fabaceae) is a suitable model for this type of comparison. It is one of the 30 most invasive plant species in the world according to IUCN [25]. In its native range, the Atlantic face of western Europe, the climate is oceanic and temperate and gorse populations are always found at the sea level (below 300 meters). The species is considered as invasive in many parts of the world at different latitudes, including New Zealand, Australia, South and North America (Chile, Colombia, California, Oregon... ) and tropical islands (Hawaii, Reunion), in altitudes that vary from zero to 4000 meters ([25] and pers). obs. Introduced populations had thus to adapt to a wide range of climates. In Europe, gorse is associated with several pathogens and herbivores, of which the specific seed-eating herbivores, the weevil *Exapion ulicis* and the moth *Cydia succedana*, are the most harmful, since they can infest 90% of a plant's pods [26]. In the invaded range, *U. europaeus* was initially introduced without any natural enemies. Some seed predators were later introduced for biological control [27], but in all the invaded areas, gorse experienced a release of selective pressures induced by seed predators for over a century. Previous studies have shown that in the native range, seed predation occurs in spring and is reduced by a genetically-based polymorphism of flowering strategy: long flowering individuals that flower in both winter and spring partly escape seed predation in time, while short flowering individuals that flower in spring reduce seed predation through mass bloom and predator satiation [28]. This polymorphism of strategy is present within all gorse populations studied and maintains a high genetic diversity for reproductive phenology (implied in escape in time), and for pod density and plant size (implied in predator satiation) [29]. As a consequence, the coexistence of these two strategies induces strong genetic correlations between these traits and infestation rates [29]. Therefore, one can expect that in the absence of selective pressure induced by seed predators, not only the trait means, but also the strength of genetic correlations among traits, may have evolved in the invaded regions.

While gorse is a good model to explore the evolution of trait means and trait correlations, it is however not suitable to produce large experimental samples: this big perennial shrub flowers at the age of three, and reaches four meters high and two meters wide. This clearly limits the number of plants that can be cultivated in an experimental garden. Since most studies were performed in annual herbaceous plants, the study of such perennial shrubs is however needed to enlarge our understanding of the invasion process. Furthermore, we knew from previous studies [28], [29] that population differences and trait correlations were so strong that they can generate significant effects even with a low number of individuals.

The aim of the present study was to explore the potential evolutionary changes that occurred in gorse invasive populations in the absence of natural enemies. We focused on seed predators, but we also considered predators that attack vegetative parts, when present. Our goals were (i) to test whether plants from invaded regions have an increased growth, reproduction, and susceptibility to seed predators compared with gorse from native regions (EICA hypothesis), (ii) to compare other life history traits related to environmental adaptation and strongly associated with susceptibility to seed predators in the native range (growth pattern and reproductive phenology), and (iii) to explore the evolution of the correlations among these traits in native and introduced regions. For this purpose we used a common garden in which gorse plants from two native regions and two invaded regions were randomly grown, and measured their trait means and trait correlations. The results were interpreted in regard of previous knowledge on gorse and theoretical expectations.

## Materials and Methods

### Study species

The common gorse *Ulex europaeus* is a spiny hexaploid shrub. It lives up to 30 years, and its adult height varies from 1 to 4 m. Plants begin to flower during their third year. Flowers are hermaphrodite and contain 10 stamens and an ovary with up to 12 ovules, enclosed in a carina. They are pollinated by large insects such as honeybees or bumblebees [30], [31]. In Europe, the peak of flowering is in spring, but there is a genetically based polymorphism of flowering phenology, with the co-existence of long-flowering individuals that flower from autumn to spring and short-flowering individuals that only flower in spring [28], [29]. Seed dispersal is primarily by ejection from the pod. Gorse seeds are very long-lasting, and may germinate over a period of up to 30 years [32].

In Europe, the most common and harmful herbivores attacking adult gorses (that are thus used for biological control), are two specific seed-eaters: the weevil *Exapion ulicis* (Coleoptera: Apionidae) [33], [34], and to a lesser extent the moth *Cydia succedana* (Lepidoptera: Tortricidae) [33]. *Exapion ulicis* is univoltine and lays its eggs in spring inside young pods. Larvae develop at the expense of seeds, and adults emerge about two months later at pod dehiscence. The larvae of *E. ulicis* can be attacked by a parasitoid wasp, *Pteromalus sequester* (Hymenoptera: Pteromalidae). Vegetative parts of adult gorses are attacked by other predators or parasites, of which the most common in the study area are the aphid *Aphis ulicis* (Homoptera: Aphididae), the spider mite *Tetranychus lintearius* (Acari: Tetranychidae), and the rust fungus *Uromyces genistae-tinctoriae* (Pucciniaceae). Young seedlings are devoid of thorns and are attacked by several generalist herbivores (e.g. rabbits and slugs).

In the invaded range, *U. europaeus* was initially introduced without its natural enemies, and is not attacked by local seed predators. Some seed predators from the native range were however introduced later for biological control. In New Zealand, *U. europaeus* was present before 1835, while *E. ulicis* was introduced in 1931 and *C. succedana* in 1992 [35]. In Reunion Island, there is no biological control program, and gorse plants still have no natural enemies.

### Experimental design

We compared plants from two native regions (Brittany, France and Scotland, UK) with plants from two invaded regions (Reunion Island, Indian Ocean and New Zealand, Pacific Ocean). In these regions, gorse was introduced by Europeans: Reunion Island was initially colonized by the French (and Brittany is the region of France where most gorse populations are located) while New Zealand was initially colonized by the Scots (and Scotland is a region that exhibits large gorse populations).

Three populations were sampled in each region (Table 1). Seeds were collected on an individual basis between 1999 and 2005, and stored at 4°C. No specific permits were required for seed collection. *Ulex europaeus* is not endangered, neither protected in none of the regions sampled: it is invasive in Reunion and New Zealand, and is an abundant native in Scotland and Brittany. The collection of its seeds does thus not require any specific authorization in any of these regions. None of the locations were privately owned. Two were situated in protected Areas and the corresponding authority was informed of our research program and did support our field work. These are The Conservatoire du Littoral in Brittany (contact: Mr. Denis Bredin) and the National Forest Office in Reunion (contact: Mr. Julien Triolo).

**Table 1:** Main characteristics of the gorse populations sampled.

Region		Location	ID	Latitude	Longitude	Elevation (m)
**Native range**						
Brittany		Cap de la Chèvre	BCC	48.1°N	04.5°W	0
		Château de Vaux	BCV	48.0°N	01.6°W	50
		Kergusul	BKE	48.0°N	03.2°W	200
Scotland		Banchory	SBA	57.1°N	02.5°W	100
		Crail	SCR	56.1°N	02.6°W	0
		Stirling	SST	56.0°N	03.9°W	300
**Invaded range**						
Reunion		Luc Boyer	RLB	21.1°S	55.6°E	1200
		Piton Maido	RMA	21.1°S	55.4°E	2200
		Piton de Brèdes	RPB	21.2°S	55.6°E	1500
New Zealand		Auckland	ZAU	37.3°S	175.1°E	0
		Christchurch	ZCH	43.6°S	172.5°E	50
		Wellington	ZWE	41.3°S	174.9°E	100

In October 2006, the seeds were scarified and allowed to germinate on Petri dishes. One seed per mother plant was randomly chosen and the resulting seedlings were grown in a greenhouse (N = 265, with a mean of 22±4 individuals per population) in cell trays and then in two-litre pots. These were filled with a mix of sand (ca 15%), perlite (ca 15%), potting soil (ca 25%) and soil collected behind gorses in a natural population (ca 45%), thus containing nitrogen-fixing bacteria gorse needs to develop. Seedlings were grown for one year in the greenhouse under horticultural lighting, at a temperature ranging from 10 to 20°C. In November 2007, seedlings were transplanted in a common garden. Due to the fact that gorse plants need three years to flower, and may grow quickly high and wide, we had to keep a minimum spacing of 1.20 m between each plant and restrict the number of seedlings transplanted. Ten seedlings per population were randomly chosen and randomly transplanted in a common garden (N = 120). The common garden is located on the Campus of Rennes (Brittany, France), an area with several gorse populations nearby and prone to natural infestation by seed predators and pathogens. Some of the individuals were left out of the analyses of reproductive traits due to non-flowering, heavy rust infestation or chlorosis, which left 8 to 10 plants per population.

### Plant height and growth pattern

Vegetative growth of the one-year-old seedlings was measured in the glasshouse in June 2007. We estimated plant height, as well as the number and length of lateral shoots. The total shoot length of each individual was estimated by adding the length of lateral shoots to the length of the main axis.

Vegetative growth at two years was measured in October 2008. We measured the height and the basal area of the individuals in the common garden. To estimate basal area, we measured the plant's largest width and the width perpendicular to it, and used the formula of an ellipse.

Vegetative growth at three years was measured in October 2009. We measured plant height and the mean length of five randomly chosen shoots per plant. It was not possible to measure their width anymore, since they had grown large enough to touch each other.

### Flowering and fruiting phenologies

We monitored reproductive phenology, reproductive output, and pod infestation every two weeks from October 2008 to July 2009. The date of flowering onset corresponded with the first occurrence of flowers together with large flower buds. The date of fruiting onset corresponded with the first occurrence of ripe pods together with browning pods.

### Reproductive output

The mean number of pods per centimetre (pod density) was estimated in spring 2009 between the end of flowering and pod dehiscence. For most individuals, this occurred in late May. For each plant, we randomly chose five shoots and counted the pods on the last 30 apical centimetres of each shoot. If the chosen shoot was shorter than 30 cm, we measured its length and counted all the pods it contained. If the chosen shoot was branched on the last 30 cm, we only retained the longest branch. We then divided the total number of pods by the total shoot length measured to get the estimation of the number of pods per centimetre.

The number of seeds per pod and seed mass were estimated when all plants had produced ripe pods, *i.e.* in late June in 2009. The number of seeds per pod was estimated on ten uninfested ripe pods per individual. Seed mass was estimated on ten seeds, from at least three different ripe pods. Flat, rotten or chewed seeds were not taken into account.

### Infestation by seed predators

The infestation rates were estimated on ripe pods. At each visit between October 2008 and July 2009, we opened 30 ripe pods, when available, and noted the number of seeds and insects. A pod was considered as infested by weevils if it contained weevils at any developmental stage (from larva to adult) or weevil parasitoids. A pod was considered as infested by a moth if it contained a moth larva, or when its presence was revealed by droppings or holes. Infestation rates were estimated when at least 10 ripe pods were available.

To compare the infestation rate of pods that were all exposed to the same abundance and activity of seed-eaters, we compared pod infestation rate at the same time for all individuals, i.e. in late June in 2009, when the highest proportion of individuals was fruiting synchronously.

The presence of predators and parasites that attack vegetative parts (the aphid *Aphis ulicis*, the spider mite *Tetranychus lintearius*, and the rust fungus *Uromyces genistae-tinctoriae*) was also recorded.

### Statistical analyses

Trait-by-trait analyses were performed using the GLM procedure of SAS software [36]. The significance of each effect was determined using type III F-statistics. We first used a three-level model, in which populations were nested within regions and regions were nested within ranges (native range and invaded range). Regions were tested as a fixed effect and populations as a random effect. Whatever the variable, the range effect was never significant. We then used a two-level model, in which populations were nested within regions. This model had a lower AIC and better fits the data. Plant height in 2007, 2008 and 2009 was tested with a repeated statement, to investigate the interaction between years and populations within regions, and between years and regions. As for the other analysis, there was no range effect and the best model only included populations nested within regions. Pod infestation rates were submitted to arcsine transformation before analysis.

The correlations between traits were estimated with the CORR procedure of SAS, using Spearman's rank-order correlation coefficient, since the data were not normally distributed. The pairwise relationships between traits were investigated using partial correlations, in which untested life history traits were used as covariates. To test for differences in correlation strength between the invaded range and the native range, we wrote a nested analysis of variance model using R software [37]. Correlation coefficients were first transformed using Fisher's *z* transformation. Then, between-regions (within ranges) and between-ranges mean squares were estimated using a weighted analysis of variance on correlation coefficients, where weights were the number of couples *n* on which each correlation coefficient was estimated. Residual mean squares were computed manually using the formula for the variance of *z*, that depends only on *n*. Since we performed partial correlations, we removed one degree of freedom for each covariate used. Finally, the significance of the effects were tested using hierarchical F-ratio tests, in which the range effect was tested against variation between regions (within ranges).

## Results

### Growth

**Figure 1:**
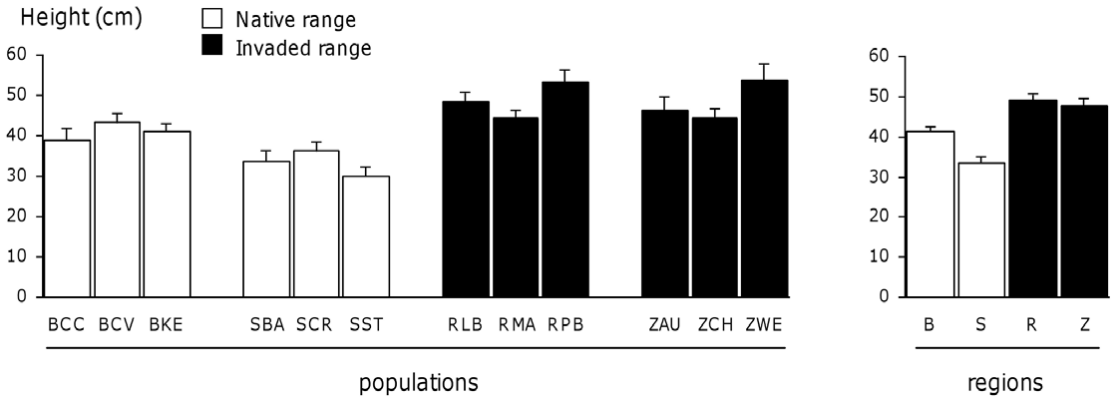
Height of one-year-old *U. europaeus* plants grown in a glasshouse. Population and regional means are given with 1 SE. N = 265.

**Figure 2:**
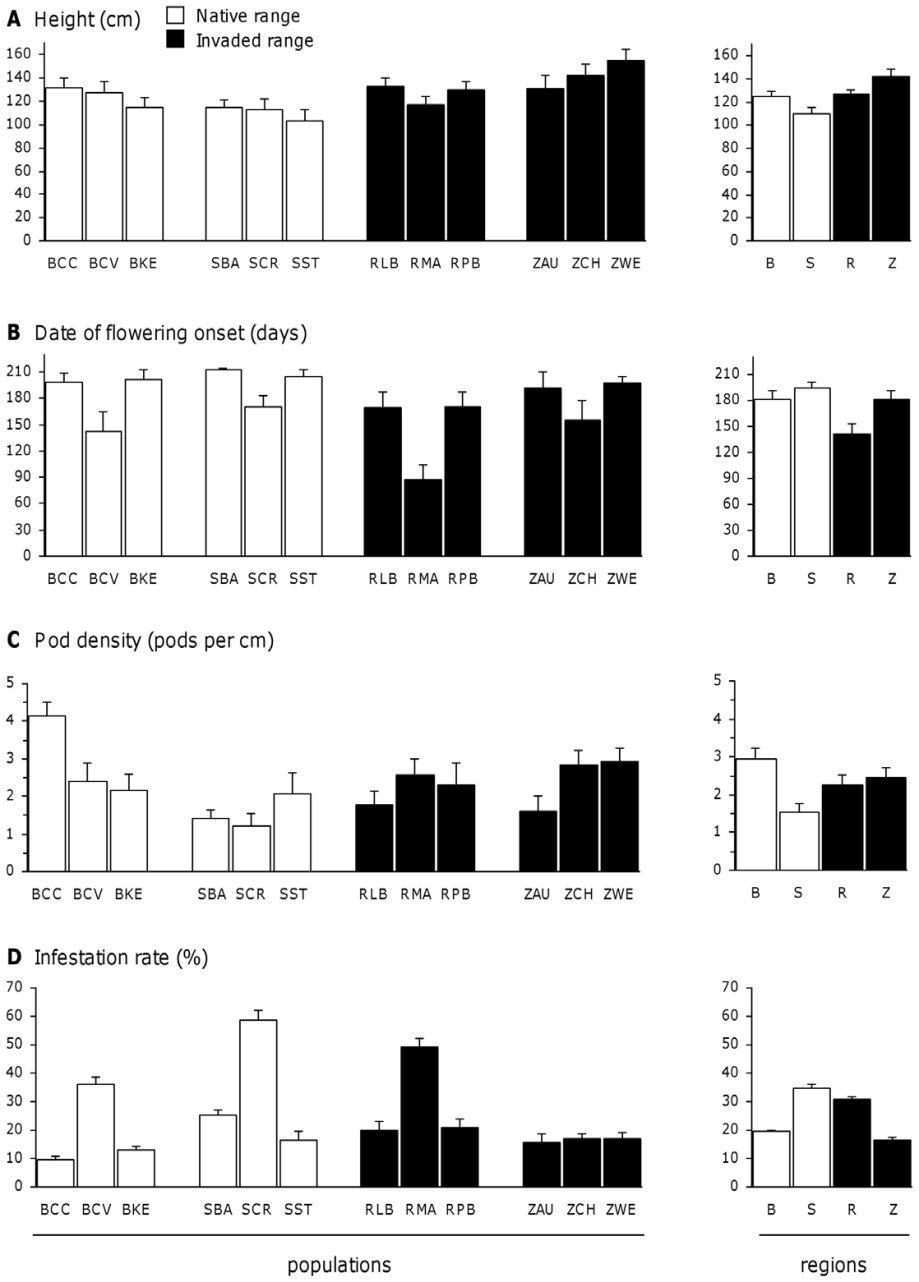
Trait means of three-year-old *U. europaeus* plants grown in a common garden. Height (A), flowering onset (B), pod density (C) and infestation rate (D). Population and regional means are given with 1 SE. Measures were done as in Table 3. N = 103 to 106 (see Table 2).

In 2007 (one-year-old plants), plant height showed significant population and region effects, the latter being stronger (Table 2). All six populations of the invaded regions were taller than the six populations of the native regions (Figure 1), and plants from the invaded regions were on average 30% taller than plants from the native regions. The number of lateral shoots and the total shoot length showed a significant population effect, but no region effect (Table 2). In 2008 (two-year-old plants), plant height still showed a significant region effect but no more population effect (Table 2). The differences between regions were less pronounced: plants from the invaded regions were on average 15% taller than plants from the native regions (Figure 2A). For basal area, there was no population effect but the region effect was significant (Table 2). Mean basal area ± SE was 1.26±0.09 m^2^ for Brittany, 0.82±0.07 m^2^ for Scotland, 1.05±0.09 m^2^ for Reunion and 0.92±0.07 m^2^ for New Zealand. In 2009 (three-year-old plants), like in 2008, plant height showed a significant region effect but no population effect (Table 2). The differences were still less pronounced: plants from the invaded regions were on average 10% taller than plants from native regions (data not shown). The mean shoot length showed a significant population effect, but no region effect (Table 2). Finally, when plant height at the three years was analysed in a repeated statement, the interaction between years and regions was significant (N = 107, F = 3.20, P<0.01), confirming that the differences among regions depended on the year. There was no interaction between years and populations (N = 107, F = 0.95, P = 0.51).

**Table 2:** Results of nested ANOVA for 18 traits of *Ulex europaeus* plants grown in a common garden.

						Populations			Regions		
		N	Mean±SD^a^			d.f.	F		d.f.	F	
**Plant height**											
June 2007 (cm)		265	42.7±13.5			8	2.29*		3	10.97**	
October 2008 (cm)		120	126.0±30.3			8	1.05		3	6.44*	
October 2009 (cm)		107	180.9±34.4			8	1.03		3	4.92*	
**Vegetative growth**											
Shoot number 2007		265	4.12±3.21			8	2.56*		3	0.57	
Total shoot length 2007 (cm)		265	100.1±52.2			8	3.03**		3	0.54	
Basal area 2008 (m^2^)		120	1.01±0.46			8	0.55		3	10.07**	
Shoot length 2009 (cm)		112	60.7±13.6			8	2.18*		3	2.67	
**Reproductive phenology** b											
Flowering onset (days)		106	173.8±55.3			8	4.93****		3	1.38	
Flowering duration (days)		106	90.8±52.0			8	3.68***		3	1.34	
Fruiting onset (days)		106	268.2±44.4			8	4.78****		3	0.59	
Fruiting duration (days)		105	36.8±44.2			8	4.68****		3	0.68	
**Reproductive output** c											
Pod density (cm^-1^)		106	2.31±1.45			8	2.80**		3	1.77	
Seeds per pod		103	3.12±0.82			8	1.74#		3	0.33	
Seed mass (mg)		105	6.48±0.89			8	1.97#		3	1.24	
**Pod infestation rates** d											
Weevils (%)		103	21.6±23.4			8	4.48****		3	0.55	
Moths (%)		103	2.53±3.67			8	0.99		3	3.67#	
Total (%)		103	25.3±25.7			8	4.54****		3	0.69	
Hymenoptera (%)		103	4.17±7.72			8	2.62*		3	0.92	

a Standard deviation of the whole sample.

b Monitored in 2008–2009, with Sept 1st 2008 taken as the first day of the reproductive season.

c Pod density was measured in May 2009; seeds per pod and seed mass were measured in late June 2009.

d Measured in late June 2009.

# P<0.10, * P<0.05, ** P<0.01, *** P<0.001, **** P<0.0001.

### Reproduction

#### Flowering and fruiting phenologies

The earliest plants began to flower in October and the latest plants in April. Whatever their date of flowering onset, all individuals flowered until spring, inducing a strong negative correlation between flowering onset and flowering duration (N = 106, R_Spearman_ =  −0.88, P<10^−4^). Flowering and fruiting onset and duration showed significant population effects, but no region effects (Table 2). The variation among populations was indeed very high and larger than the variation among regions (Figure 2B).

#### Pod and seed production

The region effect was never significant for pod density, the number of seeds per pod and seed mass (Table 2). The population effect was significant only for pod density, which exhibited a wide variability between populations (see Figure 2C). The number of seeds per pod and seed mass showed a low variability and a marginally significant population effect (Table 2).

### Infestation rates

#### Infestation of pods

The first weevil-infested pods were observed in late May, and their proportion increased continuously until the end of the fruiting season (early July). Weevil infestation rate in late June reached 22% and showed a very high population effect, but no region effect (Table 2). No region effect was detected in the other dates of observation (data not shown). Pod infestation rate by the parasitoid wasp *Pteromalus sequester* was dependent on the infestation rate by weevils, thus showing a similar pattern (Table 2). Pod infestation rate by the moth *Cydia succedana* was much lower than the infestation rate by weevils (2.5% in late June) and did not exhibit any population or region effect (Table 2). Total pod infestation rate in late June (by both weevils and moths) was mainly due to the infestation rate by weevils, and consequently showed a highly significant population effect and no region effect (Table 2, Figure 2D). These latter effects stayed the same when we used pod density and plant height, traits known to directly affect pod infestation rate, as covariates in the ANOVA (F_reg_ = 0.69, P = 0.59; F_pop_ = 4.40, P = 0.0002;).

#### Infestation of vegetative parts

In September 2008, some plants were naturally infested by the rust fungus *Uromyces genistae-tinctoriae*, and in May 2009, some plants were infested by aphids. In both cases, we recorded, for each plant, if it was infested or not, and in both cases, as for seed predators, we did not find any difference between the two invaded regions and the two native regions (data not shown). No attack by the spider mite *Tetranychus lintearius* was observed during the time of the experiment.

### Population differences

Populations within regions exhibited strong and significant differences for most traits studied, including growth, flowering phenology, reproductive output and infestation rates (Table 2). Despite the low number of populations, mean flowering onset was correlated with latitude (N = 12, R_Spearman_ = +0.59, P<0.05).

### Correlations between traits

We tested the correlations between traits in the 2008–2009 reproductive season. We retained four main traits: plant height (for growth), flowering onset (for reproductive phenology), pod density in late May (for reproductive output) and total infestation rate in late June (for seed predation). When all the individuals were pooled, most of the correlations among these traits were significant (Table 3). Further comparisons were thus performed with partial correlations in which untested life history traits were used as covariables. For example, when estimating the correlation between pod infestation rate and the date of flowering onset, pod density and plant height were used as covariates.

**Table 3:** Spearman's rank order correlation coefficients between four main traits of *Ulex europaeus* plants grown in the common garden.

	height^a^	pod density^b^	Infestation rate^c^
flowering onset^d^	−0.168#	−0.231*	−0.442***
height		0.235*	−0.218*
pod density			−0.277**

N = 103 to 106 (see Table 2).

a measured in Oct. 2008.

b measured in May 2009.

c total infestation rate (weevil + moth) in late June 2009.

d monitored between Oct. 2008 and July 2009.

# P<0.10, * P<0.05, ** P<0.01, *** P<0.001.

**Figure 3:**
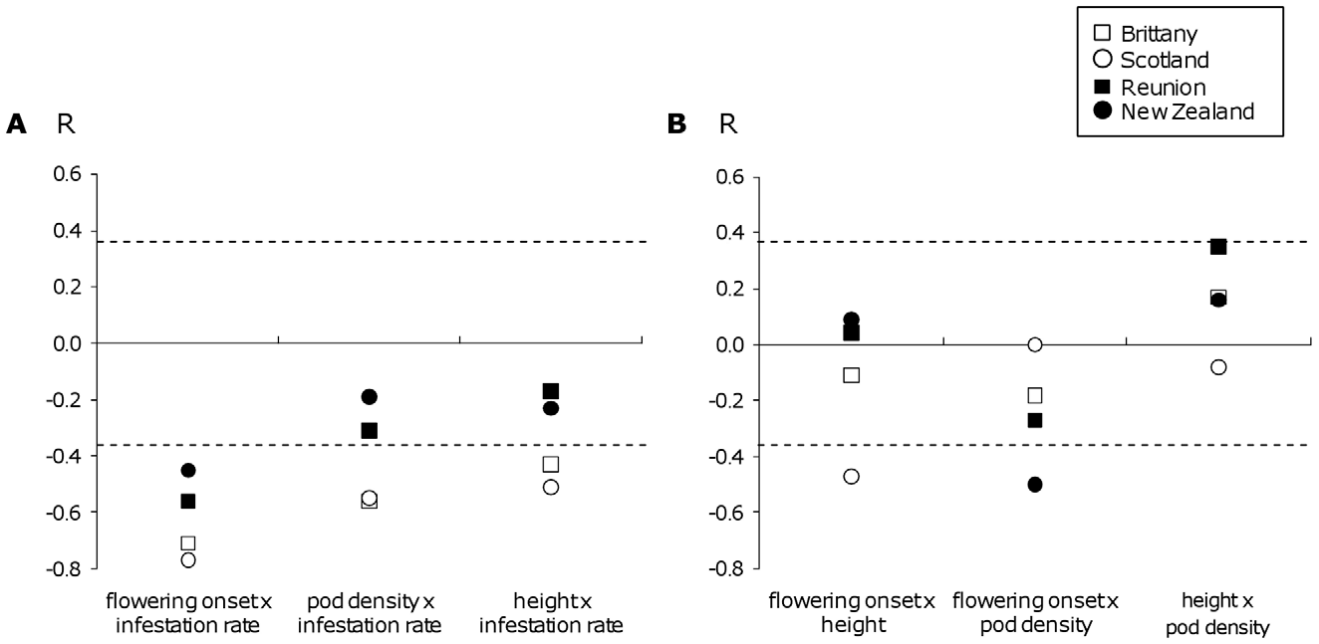
Correlations between traits of three-year-old *U. europaeus* individuals grown in a common garden. (A) correlations between infestation rate and three life history traits, (B) correlations among the three life history traits. Each point represents the Spearman's correlation coefficient for a given region. White symbols represent native regions, black symbols represent invaded regions. Measures were done as in Table 3. For each pairwise correlation, untested life history traits were used as covariables. N = 24 to 28. Dashed lines represent the 0.05 significance threshold (10^−2^ significant threshold is ±0.53, 10^−3^ significant threshold is ±0.66).

When correlations within regions were compared, the pattern obtained was different for correlations involving infestation rate (Figure 3A) and for correlations not involving infestation rate (Figure 3B). For correlations involving infestation rate, the correlation coefficients were negative and significant in the two native regions. They were also negative but weaker and rarely significant in the two invaded regions. The strength of the correlations was thus higher in the native regions than in the invaded regions (Figure 3A). The probability of observing such a consistent pattern three times can be estimated with a permutation test, where the null hypothesis is that the rank of correlation coefficients is independent of region status, and the risk alpha is the probability to obtain the observed pattern by chance. The probability for the two values of the native regions to be both higher than the two values of the invaded regions is 1/6. The probability of observing the same pattern three times is thus (1/6)^3^ = 0.0046. For correlations not involving infestation rate, correlations were low and rarely significant, and no special pattern was observed (Figure 3B). Moreover, the range effect was significant or nearly so for correlations involving infestation rate (flowering onset x infestation rate: F_1,2_ = 15.04, P = 0.06 ; pod density x infestation rate: F_1,2_ = 64.32, P = 0.02 height x infestation rate: F_1,2_ = 27.86, P = 0.03), while it was not significant for the correlations not involving infestation rate (P>0.20).

## Discussion

### Trait-by-trait analysis

According to the EICA hypothesis, we expected plants from regions of the invaded range to show an increase in growth and/or reproduction and a decrease in defence against seed predators compared to regions of the native range [16]. While the population or region effects were often highly significant, the range effect was significant for none of the traits studied. Furthermore, for all the traits studied but one, the differences between regions were not related to their range. This implies that whatever the statistical power of the range effect, the variance in these traits depended more on regions and populations of origin than on their native or invasive status.

Plant height is the only trait for which we observed a difference between the two invaded and the two native regions: plants from the two invaded regions grew taller than plants from the two native regions. Such an increased growth of plants from invaded areas was also observed in several studies performed in common gardens [12], [38], [39] where it was interpreted as a shift in resource allocation from defence to growth. In *U. europaeus*, increased growth was mainly observed on seedlings: the difference among native and invaded regions reached 30% the first year, but was reduced the second and third years. In this first year, gorse individuals were kept in a greenhouse, in an environment devoid of herbivores. Their better growth is thus in agreement with the EICA hypothesis. However, the increased growth of seedlings cannot result from reallocation of resource devoted to defence against seed predators, because individuals do not produce fruits before their third year. As a consequence, the difference in seedling height could rather result from the reallocation of resource devoted to defence against generalist herbivores that attack vegetative parts of gorse seedlings. Alternatively, it may result from maternal effects [40], or from selective pressures that were not studied here, such as competition for sunlight. Indeed, no such consistent difference between invaded and native regions was observed for the other growth parameters.

Reproductive traits did not show any significant difference among regions. This result is in agreement with the absence of difference among native and invaded regions observed in a previous study in a global comparison of seed mass, performed in natural populations of *U. europaeus* [41]. Increased fecundity of plants from the invaded range has sometimes been shown in other species (e.g. *Eschscholzia californica* [42], *Silene latifolia* [11]). However, such an increased fecundity was less often observed than increased vegetative growth [5], [17].

Finally, infestation rates were different depending on the population, but plants from the invaded and native regions exhibited similar susceptibility to the two main seed predators, *Exapion ulicis* and *Cydia succedana*. The fact that the same significance pattern remained after using pod density and plant height as covariates reveals that these traits are not the only one involved. Other features such as physical or chemical defence may be implied. Overall, these results contrast with studies on other plant species, which have often shown an increase of susceptibility to natural enemies in plants from the invaded range (e.g. in *Silene latifolia* [11], *Hypericum perforatum* [43], *Triadica sebifera* [44]). This phenomenon is however not the rule, and seems to be of importance only in some invasive species [17], [18].

Although we found that invasive gorse genotypes grew faster than native ones in their first year in the greenhouse, our results in the common garden do not support the EICA hypothesis as a major mechanism responsible for the invasiveness of *U. europaeus*. This does not exclude any involvement of EICA in this species, since growing the plants in an enemy-free environment may have revealed a reallocation of resources that could not be detected here. Still, in the classic experimental conditions used, when traits are considered independently, the native and the invaded regions were not very different, although studying more regions in each range would increase the statistical power and allow to know how general this finding is in *U. europaeus*. In any case, in our system, the variability seemed to lie mainly among populations.

### Population differences

While significant differences between regions were observed only for a few vegetative traits, populations within regions exhibited strong and significant differences for most traits studied, including growth, flowering phenology, reproductive output and infestation rates. This strong differentiation among populations was already observed in natural populations of the native regions [28], where it has been shown to be genetically determined [29]. In the invaded regions, the differences among populations observed in this study could result from genetic differentiation among source populations, drift or local adaptation.

The EICA hypothesis does not make any prediction on reproductive phenology. However, in the absence of seed predators, flowering and fruiting phenologies are expected to evolve to fit local conditions [5], independently of the constraints exerted by the avoidance of seed predation. In *U. europaeus*, flowering phenology and plant height depend on latitude and altitude [27], [45], [46]. Evidence of adaptation to altitude and latitude has been observed for vegetative growth, reproduction, and/or flowering phenology in introduced plant populations of several invasive species [9], [10], [47], [48] and, although more populations are needed to conclude, it is likely that adaptation of these traits to new environmental factors did also occur in *U. europaeus*.

Regarding infestation rates, local adaptation cannot be invoked in regions devoid of seed predators. This is notably the case in Reunion, where large differences between populations for susceptibility to seed predators are observed, even though no seed predators were ever introduced onto the island. However, pod infestation rate is an integrative measure that results from a set of factors influencing predator reproductive success, but also host choice and detection [22]. These include plant size and architecture [49], flowering phenology [50], and chemicals [51]. It is thus likely that selection acting on these traits may interact with direct selection on predator avoidance [52]. This is especially true when the different traits involved are genetically correlated, as in *U. europaeus* [29].

### Correlations between traits

Most of the traits studied here were correlated with each others, revealing again the strong relationships among them already observed in Atlan *et al.* [29]. Despite the low sample size, the correlation coefficients reached very high values and significance levels when regions were analysed separately.

For the three correlations involving the infestation rate and a life history trait (flowering onset, pod density, and plant height), the strength of the correlations was lower in the invaded regions than in the native regions. A reduction in the strength of correlations may result from a reduced diversity [53]. However, in *U. europaeus*, the diversity present in the invaded populations is very high and similar to that of the native regions, both for phenotypic traits (this study) and for neutral molecular markers (microsatellites and allozymes studies on 28 populations, Hornoy *et al.*, in prep.). Moreover, the reduction of correlation coefficients in the invaded regions was only observed in correlations involving infestation rate, suggesting that the absence of seed predation was the main causing factor reducing the strength of these correlations.

Differences between native and invaded regions thus appeared to lie in trait correlations rather than in trait means. Indeed, the trait-by-trait analysis did not reveal any clear difference between the native and the invaded ranges, and would have led to underestimating the role of the release from natural enemies in the invasive process. Such trait-by-trait analyses are often misleading when correlational selection is involved [54], which is the case in *U. europaeus* [29].

An interpretation of the differences in trait correlations observed here would need to assume that they have a genetic basis, which cannot be tested with the experimental design used in this study. However, the genetic nature of the correlations observed in the native range was ascertained in Atlan *et al.* [29] on a set of maternal families. In that previous study, correlations between family means were always greater than correlations between individuals, indicating that the small environmental differences inevitably present in a common garden, far from creating the correlations observed, were only creating noise that reduced the values of the correlations. Since the plants studied here belong to the same species, were grown in the same homogeneous conditions, randomized in the same manner, and planted in the same area as gorses studied by Atlan *et al.* [29], it seems reasonable to make the hypothesis that the phenotypic correlations observed correspond to genetic correlations. Such a hypothesis allows proposing a speculative scenario to explain the reduced correlations observed in the invaded range.

Genetic correlations can be generated by pleiotropy and/or by linkage disequilibrium [55]. In contrast to pleiotropy, that generates correlations which hold whatever the spatial scale considered, linkage disequilibrium generates correlations at the region level [56], such as those observed here. In the native range of *U. europaeus*, seed predator avoidance is achieved through a polymorphism of strategy involving phenology and growth [28], [29]. Correlational selection acting on these life history traits could thus generate strong linkage disequilibria and maintain a high level of polymorphism [21], [57].

During the invasive process, the selective pressure induced by seed predation disappeared, so that recombination and segregation could reduce the linkage in a few generations [58]. When this occurred, the polymorphic traits could have been selected to meet local conditions regardless of their consequences for susceptibility to seed predators, providing *U. europaeus* with new ecological potentialities. Interestingly, in New Zealand, where the weevil *E. ulicis* has been introduced in 1931, correlations between infestation rate and life history traits were still much lower than in native regions. Thus, the reintroduction of a biological agent was not sufficient to recreate the defence strategy observed in the native range, at least on such time scale. It would be interesting to study more invaded regions with or without biological control to see how general this relaxation of genetic correlations is in *U. europaeus*, and how long it takes for them to re-form. In any case, even if this phenomenon occurred only in a subset of invaded regions, it may still play an important role in the invasive potential of the species.

This scenario will remain speculative because the results obtained on gorse are not sufficient to ascertain the evolution of genetic correlations in the invaded range, and because the species is not suitable to generate the large dataset necessary to explore it further. However, by enhancing the importance of trait correlations, these results led us to propose a theoretical hypothesis that may provide new insights into the ecological and evolutionary mechanisms involved in the expansion of invasive species.

### Toward a new hypothesis?

Genetic constraints on life history traits have recently been shown to strongly influence the invasion dynamics and the range limits of introduced species [24], [59], [60]. Indeed, they can reduce the evolutionary potential of a species, despite the existence of genetic variance for traits considered [23], [61]. One of the main sources of genetic constraints are the genetic correlations resulting from correlational selection [21], [57]. As a consequence, if life history traits of a species are constrained by correlational selection for the avoidance of natural enemies, enemy release in the introduced range can relax these genetic correlations and enable more independent evolution of key life history traits.

We thus suggest that a relaxation of genetic constraints, and in particular a Relaxation of Genetic Correlations (or RGC) may follow enemy release and potentially enhance the adaptive potential of some introduced species. It may contribute to their invasive success by facilitating the optimisation of life history traits in the invaded range, as observed for flowering onset in *Silene latifolia* [11], [62], and flowering size in *Carduus nutans* [63]. It may also contribute to explain the niche shift or niche expansion observed in many invasive species, such as *Ulex europaeus* or *Centaurea maculosa* [64].

The RGC hypothesis takes into account the fact that the evolvability of life history traits depends not only on their genetic variability, but also on the genetic links among the traits [23], [65]. Although it does not lead to an increase of competitive ability *per se*, it may facilitate local adaptation for traits relevant for the invasive success. The RGC hypothesis may be a non-exclusive alternative to the EICA hypothesis. The proposed mechanism is more general since it can involve other features than those related to growth and reproductive effort. Also, it does not necessarily require a cost of resistance to natural enemies and is not limited to negative correlations (trade-offs) between traits. Finally, it may involve the relaxation of other genetic constraints than those related to natural enemies.
